# Deep learning to diagnose Hashimoto’s thyroiditis from sonographic images

**DOI:** 10.1038/s41467-022-31449-3

**Published:** 2022-06-29

**Authors:** Qiang Zhang, Sheng Zhang, Yi Pan, Lin Sun, Jianxin Li, Yu Qiao, Jing Zhao, Xiaoqing Wang, Yixing Feng, Yanhui Zhao, Zhiming Zheng, Xiangming Yang, Lixia Liu, Chunxin Qin, Ke Zhao, Xiaonan Liu, Caixia Li, Liuyang Zhang, Chunrui Yang, Na Zhuo, Hong Zhang, Jie Liu, Jinglei Gao, Xiaoling Di, Fanbo Meng, Linlei Zhang, Yuxuan Wang, Yuansheng Duan, Hongru Shen, Yang Li, Meng Yang, Yichen Yang, Xiaojie Xin, Xi Wei, Xuan Zhou, Rui Jin, Lun Zhang, Xudong Wang, Fengju Song, Xiangqian Zheng, Ming Gao, Kexin Chen, Xiangchun Li

**Affiliations:** 1 Department of Maxillofacial and Otorhinolaryngology Oncology, Tianjin’s Clinical Research Center for Cancer, National Clinical Research Center for Cancer, Tianjin Medical University Cancer Institute and Hospital, Tianjin Medical University, Tianjin, China; 2 Department of Diagnostic and Therapeutic Ultrasonography, Tianjin’s Clinical Research Center for Cancer, National Clinical Research Center for Cancer, Tianjin Medical University Cancer Institute and Hospital, Tianjin Medical University, Tianjin, China; 3 Department of Pathology, Tianjin’s Clinical Research Center for Cancer, National Clinical Research Center for Cancer, Tianjin Medical University Cancer Institute and Hospital, Tianjin Medical University, Tianjin, China; 4 Department of Ultrasonography, Weihai Municipal Hospital, Cheeloo College of Medicine, Jinan, Shandong Province China; 5 Department of Interventional Therapy, Tianjin’s Clinical Research Center for Cancer, National Clinical Research Center for Cancer, Tianjin Medical University Cancer Institute and Hospital, Tianjin Medical University, Tianjin, China; 6 Department of Ultrasonography, Affiliated Hospital of Chifeng University, Chifeng, Inner Mongolia Province China; 7 Department of Ultrasonography, Integrated Traditional Chinese and Western Medicine Hospital of Jilin City Jilin Province, Jilin, Jilin Province China; 8 Department of Ultrasonography, Dezhou Municipal Hospital, Dezhou, Shandong Province China; 9 Department of Ultrasound Room of Functions Branch, Affiliated Hospital of Hebei University, Zhangjiakou, Hebei Province China; 10 Department of Thyroid and Breast Surgery, Weihai Municipal Hospital, Cheeloo College of Medicine, Weihai, Shandong Province China; 11 Department of General Surgery, Tianjin Medical University General Hospital, Tianjin, China; 12 Department of Thyroid and Breast Surgery, Tianjin 4th Centre Hospital, Tianjin, China; 13 Department of Thyroid Surgery, Affiliated Hospital of Chengde Medical University, Chengde, Hebei Province China; 14 Department of Pathology, The Second Hospital of Tianjin Medical University, Tianjin, China; 15 Department of Ultrasonography, The Second Hospital of Tianjin Medical University, Tianjin, China; 16 Department of Thyroid, The Second Hospital of Jilin University, Jilin, Jilin Province China; 17 Department of Head and Neck Thyroid Surgery, Cangzhou Hospital of Integrated Traditional Chinese and Western Medicine of Hebei Province, Cangzhou, Hebei Province China; 18 Department of Surgery of Glands, People’s Hospital of Dingzhou, Dingzhou, Hebei Province China; 19 Department of Thyroid and Breast Surgery, Affiliated Hospital of Shaoxing University, Shaoxing, Zhejiang Province China; 20 Department of General Surgery, The Second Hospital of Hebei Medical University, Shijiazhuang, Hebei Province China; 21 Tianjin Cancer Institute, Tianjin’s Clinical Research Center for Cancer, Key Laboratory of Cancer Prevention and Therapy, National Clinical Research Center for Cancer, Tianjin Medical University Cancer Institute and Hospital, Tianjin Medical University, Tianjin, China; 22 Department of Epidemiology and Biostatistics, Key Laboratory of Molecular Cancer Epidemiology of Tianjin, Tianjin’s Clinical Research Center for Cancer, National Clinical Research Center for Cancer, Tianjin Medical University Cancer Institute and Hospital, Tianjin Medical University, Tianjin, China; 23 Department of Thyroid and Neck Cancer, Tianjin’s Clinical Research Center for Cancer, National Clinical Research Center for Cancer, Tianjin Medical University Cancer Institute and Hospital, Tianjin Medical University, Tianjin, China; 24 Tianjin Union Medical Center, Tianjin, China

**Keywords:** Machine learning, Thyroid diseases

## Abstract

Hashimoto’s thyroiditis (HT) is the main cause of hypothyroidism. We develop a deep learning model called HTNet for diagnosis of HT by training on 106,513 thyroid ultrasound images from 17,934 patients and test its performance on 5051 patients from 2 datasets of static images and 1 dataset of video data. HTNet achieves an area under the receiver operating curve (AUC) of 0.905 (95% CI: 0.894 to 0.915), 0.888 (0.836–0.939) and 0.895 (0.862–0.927). HTNet exceeds radiologists’ performance on accuracy (83.2% versus 79.8%; binomial test, *p* < 0.001) and sensitivity (82.6% versus 68.1%; *p* < 0.001). By integrating serologic markers with imaging data, the performance of HTNet was significantly and marginally improved on the video (AUC, 0.949 versus 0.888; DeLong’s test, *p* = 0.004) and static-image (AUC, 0.914 versus 0.901; *p* = 0.08) testing sets, respectively. HTNet may be helpful as a tool for the management of HT.

## Abstract (web summary)

Hashimoto’s thyroiditis (HT) is the main cause of hypothyroidism. Here the authors develop a deep learning model for diagnosis of HT on a large multi-site dataset including image and video data.

## Introduction

Hashimoto’s thyroiditis (HT) is a chronic autoimmune thyroid disease and the main cause of hypothyroidism and goiter^1,2^. It is prevalent in 20–30% of patients and 8–9 times more frequent in female versus male^2,3^. HT is most frequent in women aged between 30 and 50 but can occur in all ages^2^. HT accounts for 79.1% of total thyroiditis^4^. The pathogenesis of HT can be attributed to the interaction between genetic and environmental factors. Genetic susceptibility associated with HT includes genetic polymorphisms in major histocompatibility, immunoregulatory, thyroid-specific, and thyroid peroxidase antibody synthesis genes, whereas environmental factors include iodine intake, selenium, vitamin D, smoking, alcohol consumption, viral infection, and gut microbiota^1,2,5^. Patients with HT are typically presented with hypothyroidism, goiter, and increased thyroid peroxidase antibody level^5^. The key pathogenic features of HT are lymphocytic infiltration and fibrotic transformation of the thyroid gland^5^. The ultrasonographic manifestations of HT include hypoechogenicity, pseudonodule, and inhomogeneous parenchyma^6^. The former is attributed to the infiltration of inflammatory cell into the thyroid gland, whereas the latter two are due to fibroplastic proliferation^6^. The association between HT and thyroid nodule malignancy remains controversial. For example, there are studies that reported an increased risk association between HT and incidence of thyroid lymphoma and papillary thyroid cancer^7,8^, especially thyroid microcarcinoma^9^. Paparodis et al. reported that the increased risk of HT with differentiated thyroid cancer was only observed in euthyroid individuals and those with partially functional thyroid gland but not in fully hypothyroid subjects^10^. Grani et al. reported that the prevalence of thyroid nodule malignancy in patients with HT is not different from patients without HT^11^. Castagna et al. found that the association of HT with increased thyroid cancer is only observed in surgical series but not in cytological series^12^.

The symptoms of HT may not be overt as it progresses very slowly over years^13^. The clinical symptoms for patients with HT are chronic fatigue, nervousness, irritability, depression, and reduced exercise endurance^3,13^. The diagnosis of HT takes into account symptoms of hypothyroidism, presence of goiter, laboratory testing of thyroid-stimulating hormone (TSH), thyroid hormone (T4) level, antibody of thyroid peroxidase (anti-TPO), and thyroglobulin (anti-Tg)^2,13^. High serologic concentrations of anti-TPO and anti-Tg were reported to present in 90% and 20–30% of patients with HT^14^.

Artificial intelligence has attracted attention in the optical diagnosis of thyroid diseases. In a previous study, we developed a deep-learning model for optical diagnosis of thyroid cancer, in which the developed deep-learning model achieved comparable sensitivity and improved specificity in the diagnosis of thyroid cancer as compared with skilled radiologists^15^. Kim et al. evaluated the diagnostic performance of histogram analysis in the diagnosis of HT^16^. They used the grayscale features of thyroid images from histogram analysis and consensus interpretation of radiologists to develop a diagnostic model for HT. The developed models achieved an area under the curve ranging from 0.555 to 0.654^16^. Acharya et al. achieved an accuracy of 80% in the diagnosis of HT with 100 normal and 100 HT-affected ultrasound thyroid images via analyzing grayscale features such as texture, Gabor wavelet, entropy, etc.^17^. In a later study, Acharya et al. tested an ensembled model with four classifiers developed with grayscale features extracted from 526 ultrasound images in the diagnosis of HT, achieving an accuracy of 84.6%^18^. However, all these studies are limited by sample size and lack of external verification.

The purpose of this study is to develop a deep-learning model HTNet as a triage tool for automatic diagnosis of HT. We used pathological examination as the gold-standard diagnosis of HT in the development of HTNet. All subjects in the training and testing sets have pathological examination reports. HTNet was developed with by far the largest number of samples and comprehensively evaluated on internal- and external-testing sets.

## Results

### Data characteristics

**Fig. 1:**
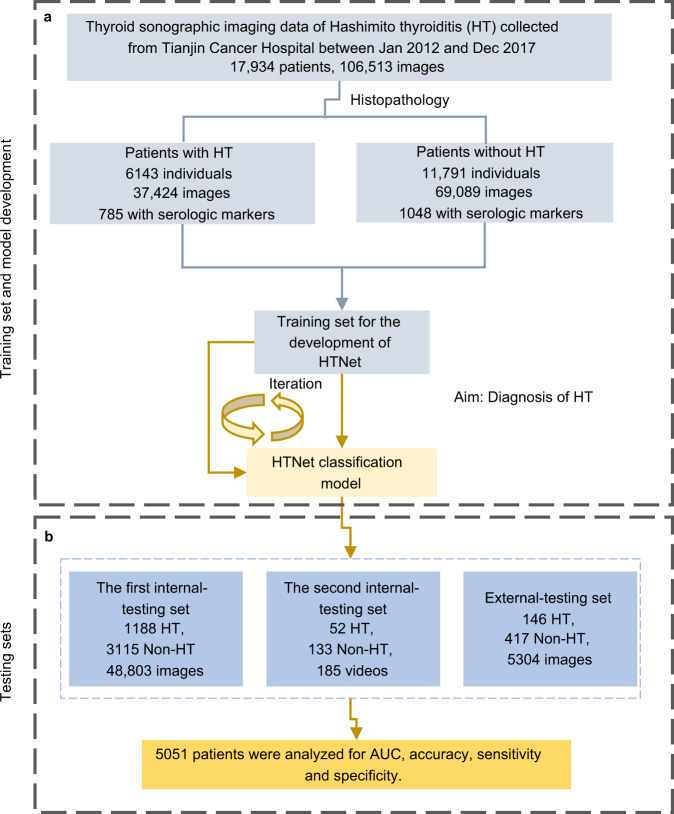
A flowchart depicting the development of HTNet. **a** Data curation and development of HTNet. **b** Evaluation of HTNet on testing sets. HT Hashimoto thyroiditis. All individuals in the training and testing sets have pathological reports for the determination of the ground truth of HT.

Between January 1, 2012, and December 15, 2017, 106,513 images from 17,934 individuals obtained from Tianjin Cancer Hospital were used as a training set after excluding 13,304 images that were not captured on the thyroid gland. The training set consisted of 6143 individuals (37,424 images) affected by HT and 11,791 controls (69,089 images). The first internal-testing set consisted of 48,803 images from 4303 individuals obtained from Tianjin Cancer Hospital between January 1, 2018, and March 28, 2019, after excluding 7655 images not captured on the thyroid gland. The second internal-testing set consisted of 185 videos from 185 individuals obtained from Tianjin Cancer Hospital collected between April 1, 2021, and May 10, 2021. The external-testing set consisted of 5304 images from 563 individuals obtained from Weihai Municipal Hospital. The baseline characteristics of the training set and three testing sets are shown in Table 1. In the training set, 34.3% (6143/17,934) of individuals were affected by HT. HT is 10 times more prevalent in female versus male. Among six serologic markers examined, Tg is lower in patients with HT than those without HT, whereas anti-Tg and anti-TPO were higher in patients with HT versus those without HT. A baseline characteristics table summarizing the clinical features of training and testing sets is provided in Table 1. A flowchart depicting the procedures to develop HTNet is provided in Fig. 1.

**Table 1:** Baseline characteristics.

Clinical feature	Training set (*n* = 17,934)		Internal-testing set 1 (*n* = 4303)		Internal-testing set 2 (*n* = 185)		External-testing set (*n* = 563)	
Disease	Patients with HT	Patients without HT	Patients with HT	Patients without HT	Patients with HT	Patients without HT	Patients with HT	Patients without HT
Patient number (*n*, %)	6143 (34.3)	11791 (65.7)	1188 (27.6)	3115 (72.4)	52 (28.1)	133 (71.9)	146 (25.9)	417 (74.1)
Age (median, IQR)	46 (18)	45 (16)	44 (17)	45 (17)	40 (24)	41 (14)	50 (18)	51 (16)
Sex (*n*, %)								
Male	556 (9.1)	3243 (27.5)	140 (11.8)	898 (28.8)	4 (7.7)	48 (36.1)	10 (6.8)	101 (24.2)
Female	5587 (90.9)	8548 (72.5)	1048 (88.2)	2217 (71.2)	48 (92.3)	85 (63.9)	136 (93.2)	316 (75.8)
Serologic marker (median, 95% CI)								
Tg (ug/L)	2.81 (0.10–62.77)	12.0 (0.57–184.89)	3.68 (0.15–100.42)	10.49 (0.53–99.17)	3.26 (0.04–81.230	16.85 (1.77–135.20)	NA	
Anti-Tg (IU/mL)	7.60 (0.92–1365.77)	0.92 (0.92–48.6)	15.74 (1.10–799.0)	7.26 (0.97–903.78)	234.0 (14.59–3521.63)	11.0 (10.0–265.5)	NA	
Anti-TPO (IU/mL)	22.34 (0.318–994.0)	0.79 (0.25–191.67)	15.92 (0.30–765.33)	0.91 (0.26–126.48)	65.5 (9.0–492.2)	9.0 (9.0–121.50)	NA	
T3 (nmol/L)	1.41 (0.97–1.95)	1.42 (1.0–1.99)	1.45 (0.94–2.07)	1.49 (1.04–2.0)	1.55 (1.19–2.32)	1.65 (1.25––2.22)	NA	
T4 (nmol/L)	99.81 (73.32–141.39)	101.0 (72.26–137.98)	95.89 (62.99–139.35)	97.55 (64.51–134.82)	89.8 (66.40–145.95)	91.2 (64.7–129.0	NA	
TSH (mIU/L)	2.29 (0.11–8.61)	1.98 (0.36–6.0)	2.33 (0.12–10.80)	2.09 (0.38–6.56)	2.29 (0.24–10.54)	1.91 (0.59–4.21)	NA

### High performance of HTNet with imaging data

**Fig. 2:**
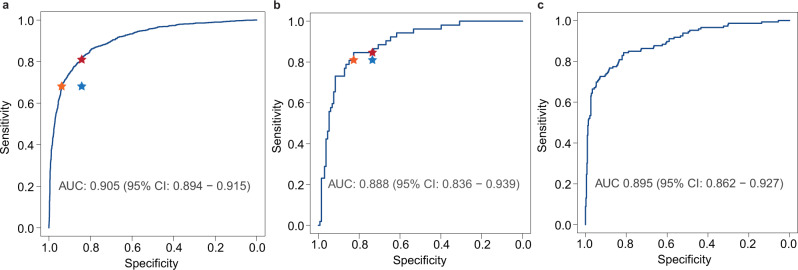
The ROC curves of HTNet on three testing sets. **a** The first internal-testing set of static images, (**b**) the second internal-testing set of video stream, (**c**) external-testing set of static images. Blue star indicates the sensitivity and specificity achieved by radiologists. The orange star indicates the specificity achieved by HTNet at the radiologists’ sensitivity. The dark red star indicates the sensitivity achieved by HTNet at the radiologists’ specificity. Area under the operating curve and associated 95% confidence intervals are included.

HTNet achieved high classification performance across these three testing sets, with AUC values of 0.905 (95% CI, 0.894–0.915) for the first internal-testing set, 0.888 (0.836–0.939) for the second internal-testing set and 0.895 (0.862–0.927) for external-testing set. The ROC curves of HTNet across testing sets are shown in Fig. 2. Across these three testing sets, the accuracy ranged from 0.823 to 0.832, sensitivity from 0.826 to 0.846, and specificity from 0.813 to 0.835. The detailed classification metrics for each testing set are provided in Table 2. On the first internal-testing set, the radiologists achieved an accuracy of 79.8% (3440/4312), sensitivity of 68.1% (809/1188), and specificity of 84.2% (2631/3124). The diagnostic performance of HTNet is not affected by the presence or absence of thyroid nodules (Supplementary Fig. 1) or by the different types of equipment used (Supplementary Fig. 2). At the radiologists’ sensitivity, HTNet achieved a specificity of 93.8%, whereas, at the radiologists’ specificity, HTNet achieved a sensitivity of 81.1%. On the second testing set, radiologists achieved an accuracy of 75.9% (151/199), sensitivity of 81.0% (51/63), and specificity of 73.5% (100/136). At the radiologists’ sensitivity, HTNet achieved a specificity of 82.7%; whereas at the radiologists’ specificity, HTNet achieved a sensitivity of 84.6%. The performance of radiologists as measured by sensitivity and specificity locates below the ROC curve (Fig. 2, left and middle panels). In addition, we used the Grad-CAM algorithm^19^ to identify image areas that most influence the decision made by HTNet. Representative thyroid ultrasound images from patients with HT together with the saliency map are shown in Supplementary Fig. 3. For the false negatives that were interpreted by radiologists derived from radiologic reports, we randomly selected 36 patients and asked three radiologists to their images. These three radiologists consensually reported that the ultrasound images from these 36 patients lack the signs manifested by HT.

**Table 2:** Classification metrics of HTNet with ultrasound images as input.

Classification metrics	Internal-testing set 1 (*n* = 4303)	Internal-testing set 2 (*n* = 185)	External-testing set (*n* = 563)
Accuracy (95% CI)	0.832 (0.821–0.843)	0.832 (0.771–0.883)	0.821 (0.786–0.851)
Sensitivity (95% CI)	0.826 (0.803–0.847)	0.846 (0.719–0.931)	0.842 (0.773–0.897)
Specificity (95% CI)	0.835 (0.821–0.848)	0.827 (0.752–0.887)	0.813 (0.772–0.849)

### Performance of HTNet by integrating serologic markers with imaging data

**Fig. 3:**
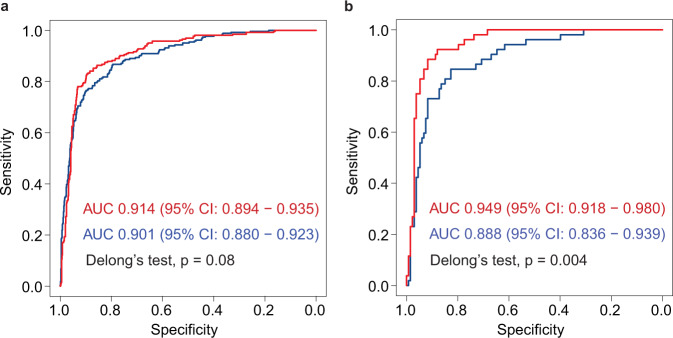
The ROC curves of HTNet with and without integration of serologic markers on static-image and video stream testing sets. **a** The first internal-testing set of static images, (**b**) the second internal-testing set of video stream. Red and blue ROC curves indicate HTNet with and without integration of serologic markers, respectively. Two-sided Delong’s test is used to test the difference between two ROC curves. Area under the operating curve and associated 95% confidence intervals are included.

In routine clinical practice, the diagnosis of HT was made by taking into account ultrasonographic features and laboratory testing of serologic markers such as TSH, anti-TPO, Tg, anti-Tg, T3, and T4. The performance of HTNet was improved by integrating serologic markers with thyroid imaging data (Fig. 3). For the element-wise summation scheme, in a subset of patients (30.0%, 945/4303) from the first internal-testing set with serologic markers, we observed that the AUC was improved from 0.901(0.880–0.923) to 0.914 (0.894–0.935), accuracy from 0.861 (0.838–0.883) to 0.877 (0.855–0.897) and sensitivity from 0.765 (0.709–0.815) to 0.830 (0.779–0.873); whereas specificities were comparable (0.896 versus 0.899). In the second internal-testing set, AUC was improved from 0.888 (0.836–0.939) to 0.949 (0.918–0.980), accuracy from 0.832 (0.771–0.883) to 0.892 (0.838–0.933), sensitivity from 0.846 (0.719–0.931) to 0.923 (0.815–0.979) and specificity from 0.827 (0.752–0.887) to 0.880 (0.812–0.930). The classification metrics achieved by HTNet with multimodal inputs via element-wise summation are shown in Table 3. Meanwhile, we found that vector concatenation scheme achieved comparable classification performance as element-wise summation on static-image set [AUC, 0.916 (0.896–0.936) versus 0.914 (0.894–0.835), *p* = 0.585] and video stream set [AUC, 0.948 (0.917–0.979) versus 0.949 (0.918–0.980), *p* = 0.766] (Supplementary Fig. 4). In addition, we observed that HTNet achieved better classification performance as compared with the random forest classifier developed with serologic markers. The ROC curve and classification metrics of the random forest classifier were provided in (Supplementary Fig. 5 and Supplementary Table 1).

**Table 3:** Classification metrics of HTNet with ultrasound images and serologic markers as input.

Classification metrics	Internal-testing set 1 (static-image, *n* = 945)	Internal-testing set 1 (static-image plus serologic markers, *n* = 945)	Internal-testing set 1 (video, *n* = 185)	Internal-testing set 1 (video plus serologic markers, *n* = 185)
Accuracy (95% CI)	0.861 (0.838–0.883)	0.877 (0.855–0.897)	0.832 (0.771–0.883)	0.892 (0.838–0.933)
Sensitivity (95% CI)	0.765 (0.709–0.815)	0.830 (0.779–0.873)	0.846 (0.719–0.931)	0.923 (0.815–0.979)
Specificity (95% CI)	0.899 (0.874–0.920)	0.896 (0.870–0.918)	0.827 (0.752–0.887)	0.880 (0.812–0.930)

## Discussions

In this study, we showed that HTNet developed with thyroid ultrasound images could achieve high performance in the diagnosis of HT on three independent testing sets from real-world settings encompassing static images and video streams. Its performance was further improved by integrating ultrasound video stream with serologic markers. HTNet was developed by far with the largest number of patients that were examined by several different types of ultrasound equipment and all patients have pathological examination as gold standard for the diagnosis of HT. The result showed that HTNet could achieve better performance as compared with ultrasound radiologists (Fig. 2, left and middle panels). HTNet may be helpful as a triage tool for the identification of HT at no extra cost. However, there is implicit cost related to the application of HTNet in clinical settings. For instance, extra time for software engineering and additional long-term maintenance are required once it was implemented clinically. This expertise is often not available in rural hospitals with scarce resources.

An accurate diagnosis of HT would be helpful for monitoring the progression of the disease and tailoring treatment regimen. Thyroid ultrasound provides a convenient and affordable way to manage thyroiditis. However, the sonographic features of HT are extremely variable and indistinguishable from the other thyroid diseases^20^. Meanwhile, interpretation of ultrasound images is often subjective, irreproducible, and operator-dependent. To address this concern, three previous studies^16–18^ proposed a computer-aided diagnostic technique that uses quantitative sonographic features and machine learning algorithm to help the diagnosis of HT, in the hope of providing objective and reproducible interpretation results. Kim et al. observed that the interobserver agreement rate was varying substantially^16^. Although they demonstrated the advantages of computer-aided diagnosis of HT, these studies were limited by small number of samples and lacked external verification^16–18^. Recently, Zhao et al. reported robust performance of an ensembled CAD-HT model via ensembling convolutional neural network models in the diagnosis of HT^21^. They found that their best model outperformed radiologists, which is consistent with our findings. Although serological markers were considered by Zhao et al., the serological markers were used to stratify individuals into different subgroups but not combined with the imaging data. Compared with these previous studies, we included by far the largest number of samples in the training set (17,934 patients) and testing sets (5051 patients). Given that hypothyroidism is mainly caused by HT, deep-learning models applied to sonographic images could provide a convenient and noninvasive method for frequently monitoring the cause of this disease. This strategy could be helpful for tailoring treatment options and delaying thyroid failure. Besides sonographic images, there are serological markers that are routinely tested in clinical settings. A deep-learning model that can take different data modalities as input is helpful for data integration and has the potential to improve diagnostic performance. The performance of HTNet was evaluated on multiple different data modalities such as static images, video stream, and combination of serological markers and imaging data. In contrast to the use of texture features manually selected by experts^16–18^, both HTNet and CAD-HT could provide an end-to-end diagnostic classification of HT directly from the raw input pixels of ultrasound images. In addition, HTNet can further take into account serological markers. However, real-time integration of sonographic images and serologic markers requires the availability of the latter. In clinical settings, there are often delays in obtaining serologic markers, thus preventing simultaneous integration of video stream and serologic markers. However, for individuals that did serologic testing ahead of sonographic examination, it is possible to integrate serologic markers during sonographic examination to obtain better diagnostic result.

Apart from the sonographic features, the levels of serologic markers such as TSH, anti-TPO, Tg, anti-Tg, T3, and T4 are helpful and routinely used in clinical practice for the diagnosis of HT and the other thyroiditis^4^. In this study, we devised a two-branched deep-learning architecture that is able to process ultrasound images and serologic markers simultaneously. Our results demonstrated that the performance of HTNet was improved considerably by integrating ultrasound images with serologic markers in the diagnosis of HT on the video testing set, whereas the improvement on the static-image testing set is marginal. The design of this two-branched deep-learning architecture is flexible in that it can be easily expanded to integrate the other types of heterogeneous data, thus making the integration of multimodal data types efficient. In the training set, the serological markers are not available for all individuals; therefore, the feature of serological markers is underrepresented and the performance of HTNet is speculatively under-estimated.

Our study has several limitations. Firstly, it is a retrospective study by nature, and the diagnostic performance of this AI system needs further investigation in prospective clinical trials. Secondly, the grade of HT was not available from the pathological examination report, therefore, we were not able to perform HT grading. Thirdly, the pathological examination reports did not have the diagnostic results for the other thyroiditis except HT, thus we were not able to perform diagnosis for the other thyroiditis such as Graves’ disease, subacute, postpartum, sporadic, and suppurative thyroiditis.

HT is the most prevalent thyroiditis and can lead to thyroid failure, reducing the quality of life. The very slow progress of HT enables a long period of time for the management of HT. There are no unique symptoms associated with HT and people with HT may not have any symptoms at the early onset, which makes early diagnosis of HT difficult. Deep-learning models applied to sonographic images could provide a convenient and noninvasive method for frequently monitoring the cause of HT. This strategy could be helpful for tailoring treatment options and delaying thyroid failure. Although the serologic markers such as anti-TPO and anti-Tg are frequently used in the diagnosis of autoimmune thyroid disease, their fluctuations are indeed associated with HT but are not a very sensitive predictor of HT^4^. The insensitivity of serologic markers in the diagnosis of HT was also demonstrated in our study (Supplementary Fig. 5). The deep-learning model developed in our study could provide a triage tool for automatic diagnosis of HT, especially in community hospitals or rural areas of China where medical resources are scarce. In addition, HTNet can also provide a second opinion that would be helpful in decision making in routine clinical practice.

The results of our study could provide improved efficiency and accuracy in a convenient way without extra cost for diagnosis of HT, especially in community hospitals where there is insufficient radiological imaging interpretation expertise. In summary, we presented a deep-learning model that could perform an automatic diagnosis of HT. Its diagnostic performance was tested on three independent testing sets. The high performance of this deep-learning model warrants further investigation in prospective clinical trials.

## Methods

### Study design and participants

We developed HTNet to diagnose HT from thyroid ultrasound images. We trained and tested this deep-learning model using thyroid ultrasound images retrospectively collected from Tianjin Cancer Hospital and Weihai Municipal Hospital. The static images extracted from the imaging database at Tianjin Cancer Hospital between January 1, 2012, and December 15, 2017, were used as a training set, static images between January 1, 2018, and March 28, 2019, as the first internal-testing set, and video data between April 1, 2021, and May 10, 2021, as the second internal-testing set. The static images from Weihai Municipal Hospital between January 1, 2017, and March 25, 2018, were used as an external-testing set. All patients in the training set and testing sets underwent pathological examination. Pathological examination reports were provided by the pathology department at Tianjin Cancer Hospital. Radiologists’ diagnosis of HT was determined from the radiologic text report. The ground truth for HT diagnosis was determined from the pathological examination report. This study was approved by the institutional review board (IRB) of Tianjin Cancer Hospital. Informed consent was exempted by the IRB because of the retrospective nature of this study. We confirmed that our research complies with the original consent of the IRB given in the treatment of these data.

### Image acquisition and preprocessing

The static images retrieved from thyroid imaging databases were in JPEG format and videos in AVI format. For a given individual, images from the entire lobe, transverse, and longitudinal view were selected by ultrasound radiologists. The ultrasound equipment from manufacturers such as Philips, Toshiba, Canon, and GE Health were used in these two hospitals to generate ultrasound images and videos. The procedures in the construction of our dataset are straightforward. We retrieved all thyroid ultrasound images from the imaging database. We linked the ultrasound image data with pathological data via the examination identity of the individual. We did not label the images and videos by annotation tool as our study is to perform diagnosis rather than lesion detection. This large dataset was made possible by a number of 16 radiologists over a long period of 10 years. In routine clinical practice, thyroid ultrasound examination was performed by one senior radiologist (≥10 years of clinical experience) and one junior radiologist (<10 years) for each individual. We excluded images that were not obtained for the thyroid gland.

### Development of the deep-learning classification model

We used the residual network^22^ for image classification. The prominent feature of residual connection is its shortcut connection that feeds the representation from preceding layers to the next layers via element-wise summation. The identity mapping via shortcut connection makes possible training very deep network without increasing training error. We trained classification network to predict HT by finetuning the classification model that we developed in our previous study^15^. The ground truth labels of HT used to train model were determined from pathological reports. We trained this model for 90 epochs by stochastic gradient descent optimizer and an initial learning rate of 0.001, momentum of 0.9, weight decay of 1.0e–4, and a minibatch of 32. The learning rate was decayed by 0.1 at the 30th and 60th epoch, respectively. We applied on-the-fly data augmentations such as random resize and crop, random horizontal flipping, random color jittering, and random erasing during training. Single-crop was used during evaluation. The classification model was developed with PyTorch (version 1.7.1) and torchvision (version 0.8.2).

### Integration of images with serologic markers in deep-learning classification model

In addition, we devised a deep-learning model that can make predictions by taking sonographic images and serologic markers obtained from laboratory testing as input. The serologic markers include TSH, anti-TPO, Tg, anti-Tg, T3, and T4. This multimodality deep-learning model consists of two parallel branches: a residual network aforementioned without the last fully connected layer and a feed-forward neural network. The residual network branch takes image as input and output a vector **F** = {**f**_**1**_, **f**_**2**_, …, **f**_**2048**_} as the representation of the input image. The feed-forward neural network branch takes the abundance of serologic markers as input and output a vector **G** = {**g**_**1**_, **g**_**2**_, …, **g**_**2048**_} as the learned feature of the input serologic markers. The element-wise summation of **F** and **G** was taken as the integrated multimodal feature **H** = **F** + **G**. Vector concatenation is an alternative method for integrating **F** and **G**, namely **H** = **[F, G]**. In this study, we investigated the performance of both element-wise summation and vector concatenation. A fully connected layer takes **H** as input and was used as the final classifier for prediction. We initialized the residual branch with the deep-learning model trained on the images aforementioned and froze their parameters. This multimodality model was trained with stochastic gradient descent for 30 epochs with a learning rate of 0.0001, momentum of 0.9 and weight decay of 1.0e–4, and a minibatch of 32. Data augmentation for images was applied exactly the same as aforementioned. We applied dropout as data augmentation for serologic markers.

### Development of traditional machine learning classification model

We employed the random forest algorithm^23^ implemented in R package randomForest^24^ to build a classifier to identify HT with the levels of six serologic markers such as TSH, anti-TPO, Tg, anti-Tg, T3, and T4. This random forest classifier was trained with 1712 samples and tested on 1130 samples. The 1712 samples were overlapped with people in the training set of ultrasound images and the later 1130 samples were overlapped with the internal-testing sets of the ultrasound images.

### Calculation of metastatic risk score

For each individual in testing set, we combined the predicted probabilities of each image or each frame of the video for that individual to calculate a score to measure the risk of HT. Specifically, for a given individual, we denoted *n* as the total number of images available from that individual, ${{{{{\boldsymbol{p}}}}}}=[{p}_{1},{p}_{2},\ldots ,{p}_{n}]$ as the probabilities of these *n* images being predicted HT. The risk score of HT $\theta$ was calculated as the average of ${{{{{\boldsymbol{p}}}}}}$. $\theta$ was used to evaluate the performance of HTNet by comparing it with the true labels obtained from pathological examination reports.

### Comparison with radiologists

We extracted the diagnosis of HT from radiologic text reports.

### Visual explanation

We used Grad-CAM algorithm^19^ to highlight the image area that most influences the decision made by HTNet.

### Statistical analysis

We used ROC curve, accuracy, sensitivity, specificity, positive predictive value (PPV), and negative predictive value (NPV) to measure the performance of HTNet and random forest classifier. The ROC curve was created by plotting sensitivity against specificity. The 95% confidence intervals for accuracy, sensitivity, specificity, PPV, and NPV were calculated by Clopper–Pearson method^25^. We plotted the ROC curve and calculated AUC with R package pROC (version 1.10.0). Statistical analysis was conducted with R software (version 4.0.3) and caret package (version 6.0-78). Random forest classifier^23^ was built with randomForest package^24^ (version 4.6-14).

### Reporting summary

Further information on research design is available in the Nature Research Reporting Summary linked to this article.

## Supplementary information

Supplementary Information

Reporting Summary

## Data Availability

Restrictions are applied to the whole imaging and serologic data of the training and testing sets, which are used with institutional permission via IRB approval for the current study, and thus are not publicly available due to patient privacy obligations. All data supporting the findings of this study are available on requests for non-commercial and academic purposes from the corresponding author X.L. (lixiangchun@tmu.edu.cn) within 10 working days.

## Code availability

The code used to train and evaluate the model is available on GitHub (https://github.com/lixiangchun/AIplus/tree/master/HTNet).

## References

[1] Liontiris MI, Mazokopakis EE (2017). A concise review of Hashimoto thyroiditis (HT) and the importance of iodine, selenium, vitamin D and gluten on the autoimmunity and dietary management of HT patients.Points that need more investigation. Hell. J. Nucl. Med..

[2] Pearce EN, Farwell AP, Braverman LE (2003). Thyroiditis. N. Engl. J. Med..

[3] Ragusa F (2019). Hashimotos’ thyroiditis: epidemiology, pathogenesis, clinic and therapy. Best. Pract. Res Clin. Endocrinol. Metab..

[4] Pedersen OM (2000). The value of ultrasonography in predicting autoimmune thyroid disease. Thyroid.

[5] Sweeney LB, Stewart C, Gaitonde DY (2014). Thyroiditis: an integrated approach. Am. Fam. Physician.

[6] Wu G, Zou D, Cai H, Liu Y (2016). Ultrasonography in the diagnosis of Hashimoto’s thyroiditis. Front Biosci. (Landmark Ed.).

[7] Resende de Paiva C, Grønhøj C, Feldt-Rasmussen U, von Buchwald C (2017). Association between Hashimoto’s thyroiditis and thyroid cancer in 64,628 patients. Front Oncol..

[8] Holm L-E, Blomgren H, Löwhagen T (1985). Cancer risks in patients with chronic lymphocytic thyroiditis. N. Engl. J. Med..

[9] Uhliarova B, Hajtman A (2018). Hashimoto’s thyroiditis – an independent risk factor for papillary carcinoma. Braz. J. Otorhinolaryngol..

[10] Paparodis R, Imam S, Todorova-Koteva K, Staii A, Jaume JC (2014). Hashimoto’s thyroiditis pathology and risk for thyroid cancer. Thyroid.

[11] Grani G (2015). Thyroid autoimmunity and risk of malignancy in thyroid nodules submitted to fine‐needle aspiration cytology. Head Neck.

[12] Castagna MG (2014). Nodules in autoimmune thyroiditis are associated with increased risk of thyroid cancer in surgical series but not in cytological series: evidence for selection bias. J. Clin. Endocrinol. Metab..

[13] American Thyroid Association. Hashimoto’s Thyroiditis Brochure. *American Thyroid Association* https://www.thyroid.org/wp-content/uploads/patients/brochures/Hashimoto_Thyroiditis.pdf (2019).

[14] Singer PA (1991). Thyroiditis. Acute, subacute, and chronic. Med Clin. North Am..

[15] Li X (2019). Diagnosis of thyroid cancer using deep convolutional neural network models applied to sonographic images: a retrospective, multicohort, diagnostic study. Lancet Oncol..

[16] Kim GR (2016). Evaluation of underlying lymphocytic thyroiditis with histogram analysis using grayscale ultrasound images. J. Ultrasound Med..

[17] Acharya UR (2013). Diagnosis of Hashimoto’s thyroiditis in ultrasound using tissue characterization and pixel classification. Proc. Inst. Mech. Eng. H..

[18] Acharya UR (2014). Computer-aided diagnostic system for detection of Hashimoto thyroiditis on ultrasound images from a Polish population. J. Ultrasound Med..

[19] Selvaraju, R. R. et al. Grad-cam: visual explanations from deep networks via gradient-based localization. In *Proceedings of the IEEE International Conference on Computer Vision* 618–626 (2017).

[20] Anderson L (2010). Hashimoto thyroiditis: part 1, sonographic analysis of the nodular form of Hashimoto thyroiditis. Am. J. Roentgenol..

[21] Zhao W (2022). Convolutional neural network-based computer-assisted diagnosis of Hashimoto’s thyroiditis on ultrasound. J. Clin. Endocrinol. Metab..

[22] He, K., Zhang, X., Ren, S. & Sun, J. Deep residual learning for image recognition. In *2016 IEEE Conference on Computer Vision and Pattern Recognition (CVPR)* 770–778 (IEEE, 2016). 10.1109/CVPR.2016.90

[23] Breiman L (2001). Random forests. Mach. Learn..

[24] Liaw A, Wiener M (2002). Classification and regression by randomForest. R. N..

[25] Newcombe RG (1998). Two‐sided confidence intervals for the single proportion: comparison of seven methods. Stat. Med..

