# Supplementary material for: Deep learning to diagnose Hashimoto’s thyroiditis from sonographic images
Source: Nat Commun. 2022 Jun 29;13:3759. doi: 10.1038/s41467-022-31449-3 (PMC9243092; doi:10.1038/s41467-022-31449-3)
Supplement: Supplementary file 1 — Supplementary Information [file 41467_2022_31449_MOESM1_ESM.pdf]

Title: Deep learning to diagnose Hashimoto's thyroiditis from sonographic images

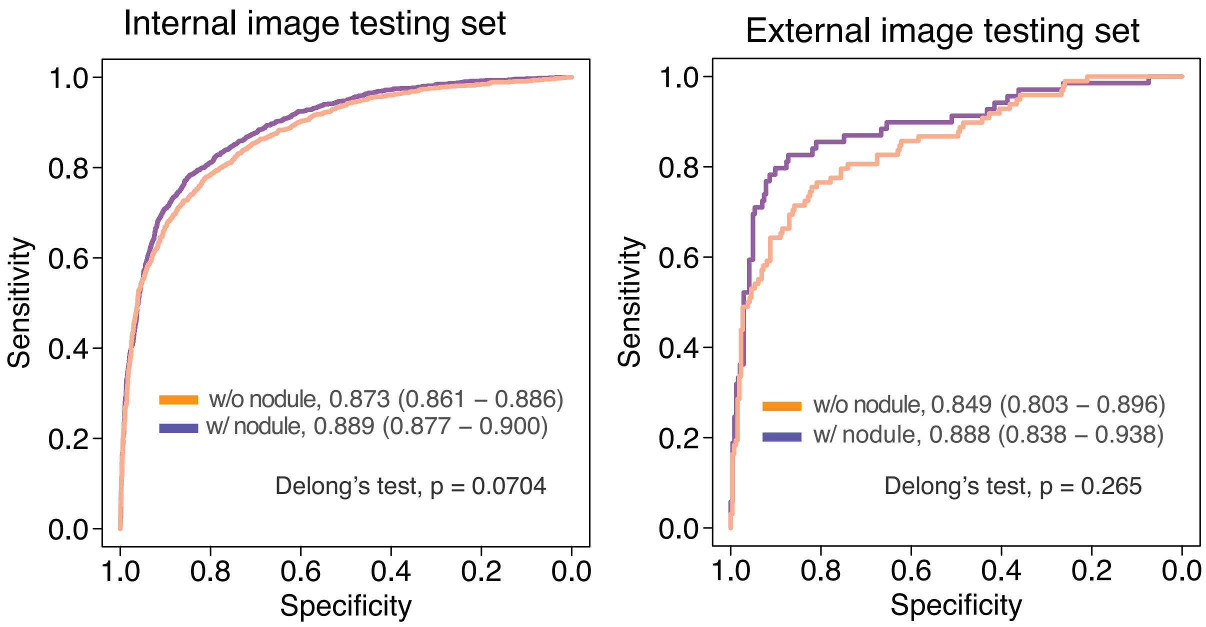

Supplementary Fig. 1. The ROC curve and AUC of HTNet on the internal- and external-testing sets on image sets with and without nodules. Area under the operating curve and associated 95% confidence intervals are included. Two-sided Delong's test was used to evaluate the difference between two ROC curves. w/o, without; w/, with.

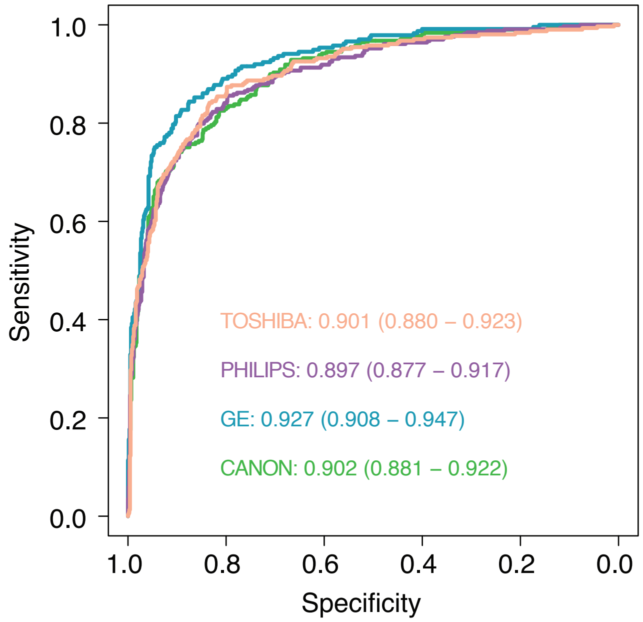

Supplementary Fig. 2. The ROC curves of HTNet on image sets stratified by manufacturers. Area under the operating curve and associated 95% confidence intervals are included.

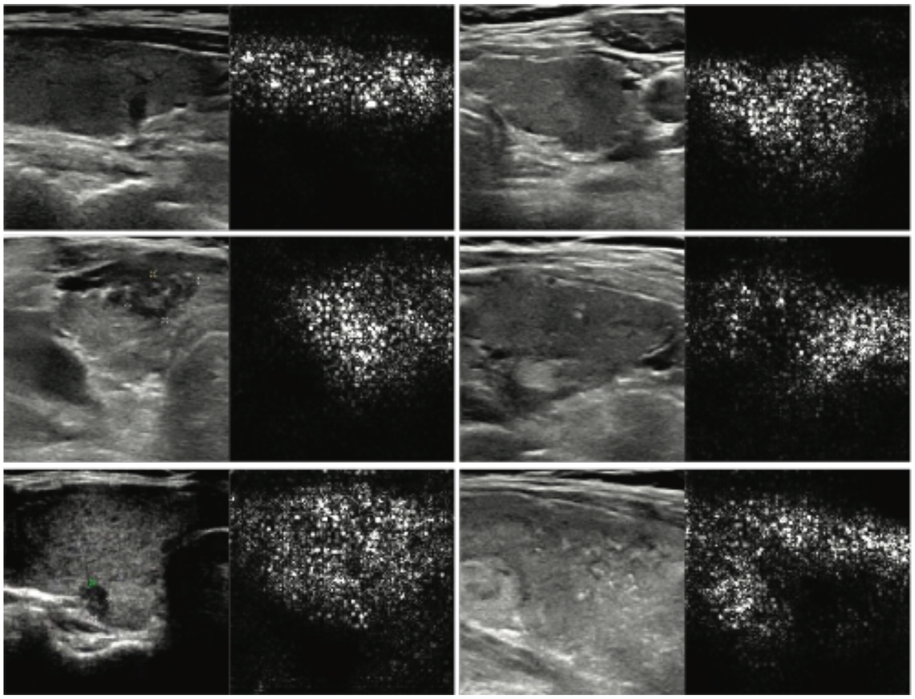

Supplementary Fig. 3. Exemplified class activation maps.

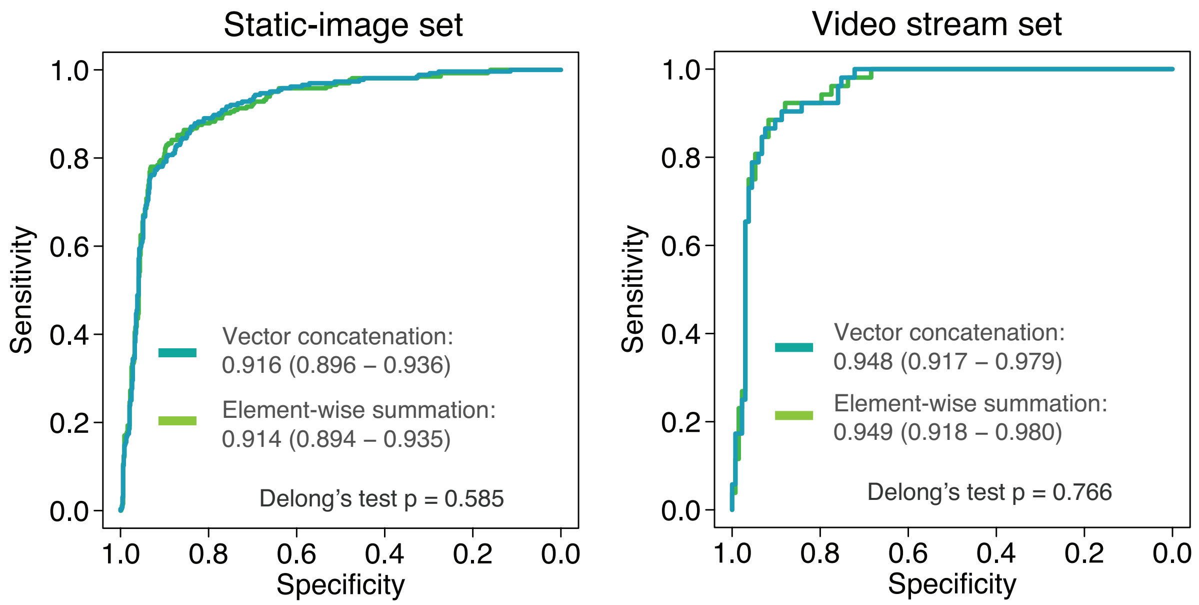

Supplementary Fig. 4. The ROC curves of HTNet for combining image features with serologic markers via element-wise summation and vector concatenation. Two-sided Delong's test was used. Area under the operating curve and associated 95% confidence intervals are included. Two-sided Delong's test was used to evaluate the difference between two ROC curves.

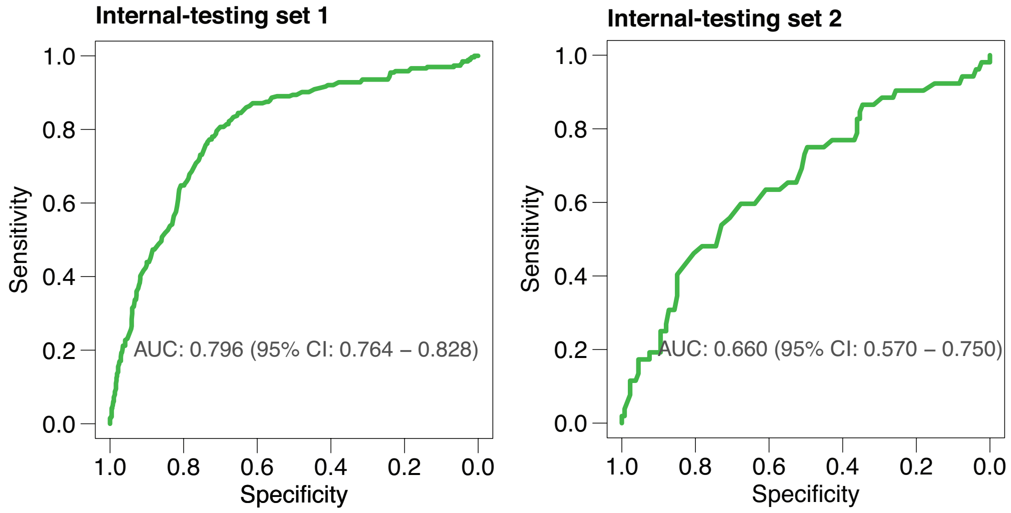

Supplementary Fig. 5. The ROC curves of random forest classifier in the diagnosis of HT on the first and second testing sets by using serologic markers.

Supplementary Table 1. Classification metrics of random forest classifier in the diagnosis of HT by using serologic markers

Classification metrics	Internal-testing set (n = 945)	Internal-testing set (n = 185)
Accuracy (95% CI)	0.730 (0.701 - 0.758)	0.654 (0.581 - 0.722)
SN (95% CI)	0.807 (0.754 - 0.853)	0.596 (0.451 - 0.730)
SP (95% CI)	0.700 (0.664 - 0.735)	0.677 (0.590 - 0.755)
PPV (95% CI)	0.511 (0.462 - 0.560)	0.419 (0.305 - 0.539)
NPV (95% CI)	0.903 (0.875 - 0.927)	0.811 (0.725 - 0.879)
Kappa	0.431	0.242
F1	0.626	0.492
