# Supplementary material for: Deep learning to diagnose Hashimoto’s thyroiditis from sonographic images
Source: Nat Commun. 2022 Jun 29;13:3759. doi: 10.1038/s41467-022-31449-3 (PMC9243092; doi:10.1038/s41467-022-31449-3)
Supplement: Supplementary file 2 — Reporting Summary [file 41467_2022_31449_MOESM2_ESM.pdf]

nature portfolio

Corresponding author(s): Xiangchun Li

Last updated by author(s): Apr 17, 2022

Reporting Summary

Nature Portfolio wishes to improve the reproducibility of the work that we publish. This form provides structure for consistency and transparency in reporting. For further information on Nature Portfolio policies, see our Editorial Policies and the Editorial Policy Checklist.

Statistics

For all statistical analyses, confirm that the following items are present in the figure legend, table legend, main text, or Methods section.

n/a	Confirmed	
☐	☒	The exact sample size (*n*) for each experimental group/condition, given as a discrete number and unit of measurement
☐	☒	A statement on whether measurements were taken from distinct samples or whether the same sample was measured repeatedly
☐	☒	The statistical test(s) used AND whether they are one- or two-sided<br>*Only common tests should be described solely by name; describe more complex techniques in the Methods section.*
☒	☐	A description of all covariates tested
☒	☐	A description of any assumptions or corrections, such as tests of normality and adjustment for multiple comparisons
☐	☒	A full description of the statistical parameters including central tendency (e.g. means) or other basic estimates (e.g. regression coefficient) AND variation (e.g. standard deviation) or associated estimates of uncertainty (e.g. confidence intervals)
☐	☒	For null hypothesis testing, the test statistic (e.g. *F*, *t*, *r*) with confidence intervals, effect sizes, degrees of freedom and *P* value noted<br>*Give P values as exact values whenever suitable.*
☒	☐	For Bayesian analysis, information on the choice of priors and Markov chain Monte Carlo settings
☒	☐	For hierarchical and complex designs, identification of the appropriate level for tests and full reporting of outcomes
☒	☐	Estimates of effect sizes (e.g. Cohen's *d*, Pearson's *r*), indicating how they were calculated

Our web collection on statistics for biologists contains articles on many of the points above.

Software and code

Policy information about availability of computer code

Data collection	No software was used. Source code of HTNet available at https://github.com/lixiangchun/AIplus/tree/master/HTNet.
Data analysis	We developed a deep learning model called HTNet by training a ResNet on ultrasound thyroid images to differentiate Hashimoto thyroiditis from its controls. All image labels were derived from pathological examination reports. For each individual, we calculated a score by averaging all images predicted to be Hashimoto's thyroiditis from this individual. The classification performance of our model was independently validated three testing sets encompassed static images and videos, and compared with skilled ultrasound radiologists. The deep learning pipeline was developed with PyTorch (v1.7.1) and TorchVision (v0.8.2). We plotted the ROC curve and calculated AUC with R package pROC (version 1.10.0). Statistical analysis was conducted with R software (version 4.0.3) and caret package (version 6.0-78). Random forest classifier was built with randomForest package (version 4.6-14).

For manuscripts utilizing custom algorithms or software that are central to the research but not yet described in published literature, software must be made available to editors and reviewers. We strongly encourage code deposition in a community repository (e.g. GitHub). See the Nature Portfolio guidelines for submitting code & software for further information.

Data

Policy information about availability of data

All manuscripts must include a data availability statement. This statement should provide the following information, where applicable:

- Accession codes, unique identifiers, or web links for publicly available datasets
- A description of any restrictions on data availability
- For clinical datasets or third party data, please ensure that the statement adheres to our policy

The supporting data generated in this study are provided in the Supplementary Information file. The raw thyroid ultrasonographic imaging data are protected and

are not available due to data privacy laws. The authors declare that the data supporting the findings of this study are available within the paper and its supplementary information files. Restrictions apply to the availability of the training and test sets, which were used with permission for the current study, and so are not publicly available.

Field-specific reporting

Please select the one below that is the best fit for your research. If you are not sure, read the appropriate sections before making your selection.

☒ Life sciences ☐ Behavioural & social sciences ☐ Ecological, evolutionary & environmental sciences

For a reference copy of the document with all sections, see nature.com/documents/nr-reporting-summary-flat.pdf

Life sciences study design

All studies must disclose on these points even when the disclosure is negative.

Sample size	No sample-size calculation was performed. We used all the images retrieved from Thyroid Imaging Database. The sample size of the training set is 106,513, which is sufficient to finetune a deep learning model. The number of static images in two testing sets are 48,803 and 5304, respectively. The video testing set consists of 185 video.
Data exclusions	We excluded 13304 images that were not captured on the thyroid gland.
Replication	The findings were successfully validated in three independent testing sets.
Randomization	We trained and tested this deep learning model using thyroid ultrasound images retrospectively collected from Tianjin Cancer Hospital and Weihai Municipal Hospital. The static images extracted from imaging database at Tianjin Cancer Hospital between Jan 1, 2012 and Dec 15, 2017 was used as training set, static images between Jan 1, 2018 and Mar 28, 2019 as first internal-testing set, and video data between April 1, 2021 and May 10, 2021 as the second internal-testing set. The static images from Weihai Municipal Hospital between Jan 1, 2017 and Mar 25, 2018 were used as external-testing set.
Blinding	Sonographic imaging data, radiologic text reports and pathological examination reports were extracted from the database. The ground truth of Hashimito Thyroiditis was determined from pathological reports. Radiologists' diagnosis of Hashimito Thyroiditis was determined from radiological text reports. Radiologists were blinded to the occurrence of Hashimoto Thyroiditis in both the training and testing sets.

Reporting for specific materials, systems and methods

We require information from authors about some types of materials, experimental systems and methods used in many studies. Here, indicate whether each material, system or method listed is relevant to your study. If you are not sure if a list item applies to your research, read the appropriate section before selecting a response.

Materials & experimental systems

n/a	Involved in the study	
☒	☐	Antibodies
☒	☐	Eukaryotic cell lines
☒	☐	Palaeontology and archaeology
☒	☐	Animals and other organisms
☐	☒	Human research participants
☒	☐	Clinical data
☒	☐	Dual use research of concern

Methods

n/a	Involved in the study	
☒	☐	ChIP-seq
☒	☐	Flow cytometry
☒	☐	MRI-based neuroimaging

Human research participants

Policy information about studies involving human research participants

Population characteristics	The baseline characteristics was shown in Table 1. Male and female patients were included. In the training set, the Hashimoto's thyroiditis group included 9.1% of male and 90.9% of female; the non-Hashimoto's thyroiditis group included 27.5% of male and 72.5% of female. In the first testing set, the Hashimoto's thyroiditis group included 11.8% of male and 88.2% of female; the non-Hashimoto's thyroiditis group included 28.8% of male and 71.2% of female. In the second testing set, the Hashimoto's thyroiditis group included 7.7% of male and 92.3% of female; the non-Hashimoto's thyroiditis group included 36.1% of male and 63.9% of female. In the third testing set, the Hashimoto's thyroiditis group included 6.8% of male and 93.2% of female; the non-Hashimoto's thyroiditis group included 24.2% of male and 75.8% of female. The median age was 46 and 45 for individuals with and without HT in the training set, 44 and 45 in the static-image internal-testing set, 40 and 41 in the video-stream internal-testing set, 50 and 51 in the static-image external-testing set. Serologic markers collected include Tg, Anti-Tg, Anti-TPO, T3, T4, and TSH.
Recruitment	This is a retrospective study so all participants extracted from the imaging database were included.
Ethics oversight	This study was approved by the Institutional Review Board of Tianjin Medical University Cancer Institute and Hospital.

Note that full information on the approval of the study protocol must also be provided in the manuscript.
